# Phase Transition of the Bacterium upon Invasion of a Host Cell as a Mechanism of Adaptation: a *Mycoplasma gallisepticum* Model

**DOI:** 10.1038/srep35959

**Published:** 2016-10-24

**Authors:** Daria Matyushkina, Olga Pobeguts, Ivan Butenko, Anna Vanyushkina, Nicolay Anikanov, Olga Bukato, Daria Evsyutina, Alexandra Bogomazova, Maria Lagarkova, Tatiana Semashko, Irina Garanina, Vladislav Babenko, Maria Vakhitova, Valentina Ladygina, Gleb Fisunov, Vadim Govorun

**Affiliations:** 1 Laboratory of Proteomic Analysis, Federal Research and Clinical Centre of Physical-Chemical Medicine, Moscow 119435, Russia; 2 Laboratory of Proteomics, Shemyakin-Ovchinnikov Institute of Bioorganic Chemistry, Moscow 117997, Russia; 3 Department of Bioinformatics and Bioengineering, Lomonosov Moscow State University, Moscow 119234, Russia; 4 Laboratory of Cell Biology, Federal Research and Clinical Centre of Physical-Chemical Medicine, Moscow 119435, Russia; 5 Stem Cell Laboratory, Vavilov Institute of General Genetics RAS, Moscow 119991, Russia; 6 Laboratory of Post-Genomic Research in Biology, Federal Research and Clinical Centre of Physical-Chemical Medicine, Moscow 119435, Russia; 7 Moscow Institute of Physics and Technology (State University), Dolgoprudny 141700, Russia

## Abstract

What strategies do bacteria employ for adaptation to their hosts and are these strategies different for varied hosts? To date, many studies on the interaction of the bacterium and its host have been published. However, global changes in the bacterial cell in the process of invasion and persistence, remain poorly understood. In this study, we demonstrated phase transition of the avian pathogen *Mycoplasma gallisepticum* upon invasion of the various types of eukaryotic cells (human, chicken, and mouse) which was stable during several passages after isolation of intracellular clones and recultivation in a culture medium. It was shown that this phase transition is manifested in changes at the proteomic, genomic and metabolomic levels. Eukaryotic cells induced similar proteome reorganization of *M. gallisepticum* during infection, despite different origins of the host cell lines. Proteomic changes affected a broad range of processes including metabolism, translation and oxidative stress response. We determined that the activation of glycerol utilization, overproduction of hydrogen peroxide and the upregulation of the SpxA regulatory protein occurred during intracellular infection. We propose SpxA as an important regulator for the adaptation of *M. gallisepticum* to an intracellular environment.

Parasitism is one of the mechanisms of interaction of the bacteria with their hosts. However, many aspects of this phenomenon are poorly understood for most bacteria. For many years, biologists were interested in questions why each of the pathogens has a certain host, and what are the specific mechanisms of host-parasite interactions? Bacteria of the genus Mycoplasma despite they are widespread, are those with largely unknown pathogenicity mechanisms. Almost all living creatures-humans, animals, plants and fungi are the hosts of mycoplasmas, and compact genome of Mycoplasma makes it convenient model for the omics-based studies.

Members of the genus Mycoplasma (class Mollicutes) are Gram-positive bacteria, lack a cell wall and contain a small genome of 0.58–2.20 Mb. Because of their parasitic lifestyle, the mycoplasmas also have significantly fewer metabolic pathways; therefore, their survival depends greatly on their interaction with a host cell. Mycoplasmas are widespread bacteria and the latest data in the literature indicates that one of the types of fungal endobacteria belongs to Mollicutes (“Mollicutes-related endobacteria”; MRE)^1^,^2^. They were detected in the intraradical and extraradical mycelium and in the spores of arbuscular mycorrhizal fungi^3^. These findings even more extend the range of mycoplasma habitat. The study of mycoplasmas is more intriguing because these bacteria are able to persist for a long time in the host, undetected by the immune system, providing a good model for studying the transition from parasitism to endosymbiosis. In nature, such transitions are known not only for MRE but also for *Wolbachia,* for example^4^,^5^.

*Mycoplasma gallisepticum* induces severe chronic respiratory disease in chickens and sinusitis in turkeys. However, recently it has jumped to wild house finches that were previously not considered to be a host^6^,^7^, reinforcing the idea that over time, bacteria adapt to their surrounding environment and occupy new niches for life. Despite the fact that the majority of the published data claim that *M. gallisepticum* is a parietal parasite, a number of studies have shown the ability of *M. gallisepticum* to infect eukaryotic cells such as HeLa-229 and chicken embryonic fibroblasts^8^, and Vogl *et al.* showed the ability of *M. gallisepticum* to infect non-phagocytic cells such as chicken erythrocytes^9^,^10^. It has been shown that after infection, *M. gallisepticum* spreads throughout the body. In chickens experimentally inoculated via an aerosol, mycoplasma were localized in the spleen, heart, brain and kidneys^11^. The mechanism of the transition of a local infection to a systemic one is not fully understood.

In this study we have observed a striking proteomic response of *M. gallisepticum* to external conditions. In *Mycoplasma pneumonia*, protein changes have been demonstrated in response to heat shock, DNA damage and osmotic stress^12^. Although protein changes were detected, the fold-change was low. Our previous experiments did not identify significant protein changes in heat shock as well^13^. Possibly the invasion into eukaryotic cells is a more powerful stimulus for the response which affects a number of key nodes (“hot points”) in the metabolism and regulatory systems of mycoplasma leading to a transition to another state.

The study of different bacteria and their interaction with a host cell on the proteomic and transcriptomic level has not resulted in a clear understanding of which global changes are needed for bacteria^14^,^15^,^16^,^17^,^18^. In *Mycoplasma hyorhinis,* the depletion of CG-specific methylation of the genomic DNA after host cell invasion has been shown^19^. The authors assumed it is likely that variations in the CG methylation levels in the *M. hyorhinis* genome contributed to the fitness and survival of this bacterium both inside and outside of infected host cells. It has been shown for *M. gallisepticum* that upon transition to the house finch from poultry, CRISPR arrays first demonstrated the increased uptake of new spacers and a general, progressive reorganization, after which the CRISPR arrays undergo reduction^6^.

Documenting the evolutionary changes occurring in pathogens when they switch hosts is important to understand adaptation mechanisms and evolution rates^6^. In this study, we investigated the capacity of *M. gallisepticum S6* to switch to another phase state during the invasion of various eukaryotic host cells and maintain that state for several passages. For the first time, we showed that *M. gallisepticum* undergoes a systemic rearrangement in the intracellular environment that occurs at the proteomic, genomic and metabolomic levels. We propose that the SpxA protein is a global regulator of the transition to this altered state because in another stress conditions, for example, heat shock, we did not observe upregulation of this protein^13^. Thus, this study will help shed light on the mechanisms of adaptation and bacterial evolution.

## Results

### *M. gallisepticum* is capable of the intracellular infection of eukaryotic cells

The ability of *M. gallisepticum* to penetrate into eukaryotic cells was studied by infecting three different cell lines: HeLa-229 cervical cancer cells, chicken erythroblast cells (HD3) and mES murine embryonic stem cells. The cell cultures were regularly checked for mycoplasma contamination by culture and PCR. To exclude the effect of possible population variations, a clonal mycoplasma culture was used.

**Figure 1:**
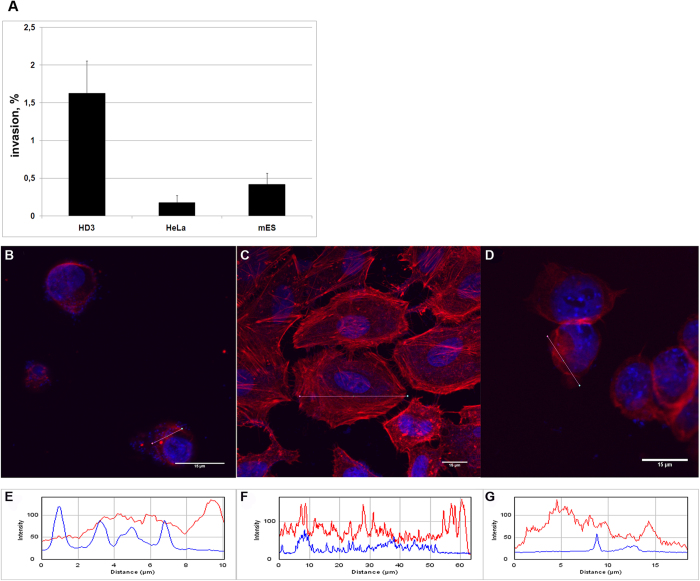
Invasion of *M. gallisepticum* into three eukaryotic cell lines under acute infection conditions. (**A**) The percentage of invasion was calculated by dividing the CFU value obtained after gentamicin treatment with the CFU value of total mycoplasmas added for infection and multiplied by 100. The data represent the mean (±SD) of three independent experiments performed in triplicate. (**B–D**) Fluorescence was visualized with confocal microscopy. Alexa Fluor 568 phalloidin fluorescence showing eukaryotic cell F-actin stained red, DAPI fluorescence showing cell nuclei and mycoplasmas stained blue. Scale bars, 15 μm. (**B**) HD3 cells, (**C**) HeLa-229 cells, (**D**) mES cells. In addition, the intensity distributions of DAPI and phalloidin confirm the intracellular localization of *M. gallisepticum* in HD3, HeLa and mES cells, which is further illustrated by the RGB profiles seen in (**E–G**), respectively. The pink line in (**B–D**) represents the location of RGB profile analysis.

Eukaryotic cell lines were infected by *M. gallisepticum S6* at a ratio of 1:1,000 respectively. Eukaryotic cells were cultivated with the mycoplasma for 24 hours (acute infection), 19 days or 7 weeks (chronic infection). We applied the standard gentamicin invasion assay used in many mycoplasma invasion studies^8^,^9^,^20^,^21^,^22^,^23^. After all types of cultivation, the cells were treated with 600 μg/ml of gentamicin to eliminate 100% of the extracellular mycoplasma and only intracellular mycoplasma survived. The dose of gentamicin for complete *M. gallisepticum* elimination was selected using CFU test to produce zero colonies. The actual concentration of gentamicin exceeded minimal inhibitory concentration 6-fold. The conditions of gentamicin treatment in CFU test, including culture medium and incubation time, were exactly the same as for the experiment with eukaryotic cells. After gentamicin treatment the cell lines were subsequently plated on semiliquid agar to identify viable intracellular bacteria by CFU counts in parallel with control *M. gallisepticum* strain. Growth of *M. gallisepticum* colonies on the semiliquid agar demonstrated the ability of the bacteria to penetrate into eukaryotic cells. As a negative control, we used an eukaryotic cell suspension with culture media for *M. gallisepticum*. The invasion frequency was measured as a ratio of the CFU number after incubation to the CFU number in the initial culture. Figure 1A shows the number of colonies that grew after plating the infected cells on semiliquid agar for all three cell lines under conditions of acute infection. Mycoplasma clones that were isolated after invasion were used for a second round of chronic infection in HD3 cells. Intracellular mycoplasma isolated from eukaryotic cells after gentamicin treatment and semiliquid agar stadium will be referred to hereafter as MIEC. The colonies of either MIEC or control mycoplasma grown on agar were picked out and cultivated in liquid medium until exponential phase. We used confocal microscopy to confirm the ability of *M. gallisepticum* to penetrate into the eukaryotic cells. Confocal micrographs of HD3, HeLa and mES cells infected for 24 h demonstrate the presence of intracellular *M. gallisepticum* cells (Fig. 1B–G, Supplementary Figs S1–S3). To elucidate the molecular basis of the host-pathogen interactions, we performed a metabolomic, genomic and proteomic analysis of the MIEC.

### 2D-DIGE demonstrates global proteome reorganization of *M. gallisepticum* during intracellular infection

**Figure 2:**
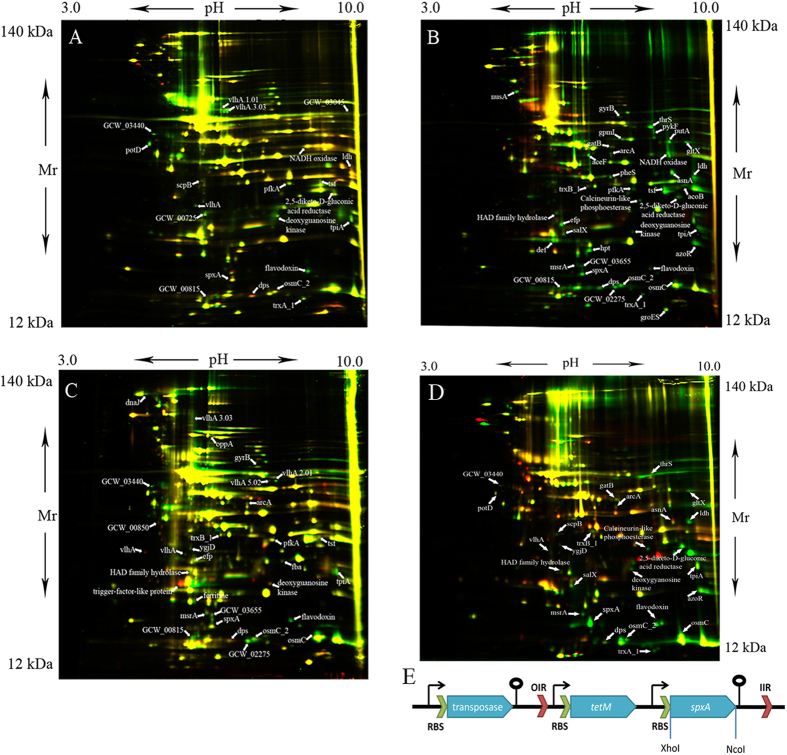
2-D DIGE analysis of *M. gallisepticum* isolated from HD3 cells and overexpressed spxA. (**A**) *M. gallisepticum* after 24 h of infection (Cy3, green) and laboratory strain *M. gallisepticum S6* (Cy5, red). (**B**) *M. gallisepticum* after chronic infection (19 days) (Cy3, green) and laboratory strain *M. gallisepticum S6* (Cy5, red). (**C**) *M. gallisepticum* after chronic infection (7weeks) (Cy3, green) and laboratory strain *M. gallisepticum S6* (Cy5, red). (**D**) SpxA-overexpressing *M. gallisepticum* (Cy3, green) and laboratory strain *M. gallisepticum S6* (Cy5, red). (**E**) Schematic construction of a transposon vector for overexpression of the *spxA* gene. RBS-ribosome binding site; OIR and IIR– inverted repeats.

*M. gallisepticum* isolated from the cell lines was compared with a control laboratory strain using two-dimensional electrophoresis with differential staining. The cells obtained after acute infection (24-hour infection) and chronic infection (19 days or 7 weeks of infection) were used for a comparative proteomic profile. The mycoplasma of the same passage cultivated in semiliquid and liquid media was used as a control. Figure 2A–C shows reference 2D-maps that compare the proteomic profiles of *M. gallisepticum* cells obtained after 24 h of infection (Fig. 2A), 19 days (Fig. 2B) and 7 weeks of chronic infection (Fig. 2C) with the proteomic profile of the reference laboratory strain *M. gallisepticum S6*.

**Figure 3:**
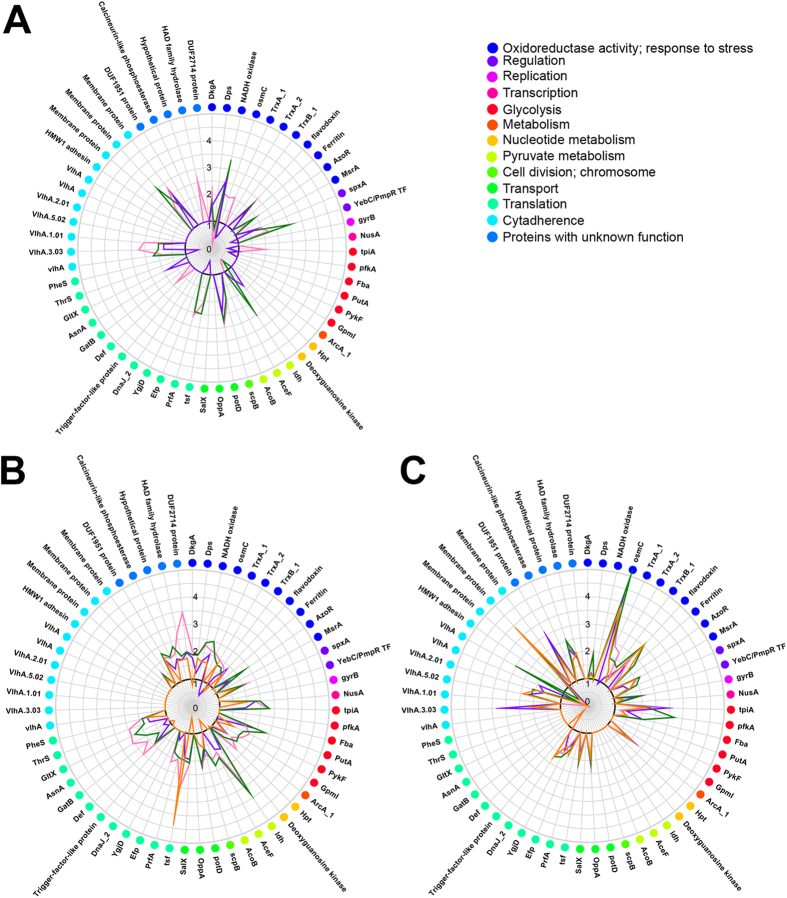
Radar chart of proteins in *M. gallisepticum* isolated from three different cell lines after acute infection and chronic infection in HD3 cells compared with the control laboratory *M. gallisepticum S6* strain using differential 2D gel electrophoresis. *M. gallisepticum* initially isolated from HD3 cells–green line, *M. gallisepticum* initially isolated from Hela cells–violet line, *M. gallisepticum* initially isolated from mES cells–pink line, *M. gallisepticum*–orange line. The black circle represents the threshold (the ratio of the protein level in the intracellular mycoplasma relative to the control strain is (1). Values based on the ratio of the fluorescence intensity for the channels Cy3/Cy5 were counted using the PDQuest software package (Bio-Rad). MIEC marked by green Fluorescent dye (Cy3), a control laboratory strain of *M. gallisepticum S6*-red (Cy5). Detailed information for the proteins can be obtained in Table S1. (**A**) 24-h acute infection. (**B**) 19-days chronic infection. (**C**) 7-weeks chronic infection.

After 24 hours of infection, we observed changes in the expression of proteins that had oxidoreductase activity and proteins involved in oxidative stress protection (Fig. 3A, Supplementary Table S1), including 2,5-diketo-D-gluconic acid reductase, OsmC, Dps, flavodoxin and TrxA_1 (thioredoxin). We also observed the upregulation of a global regulator SpxA as well as a cell division protein ScpB, transport protein PotD and NADH oxidase. In addition, we observed an increase in the amount of the two major VlhA hemagglutinins (GCW_03350, GCW_01940).

**Figure 4:**
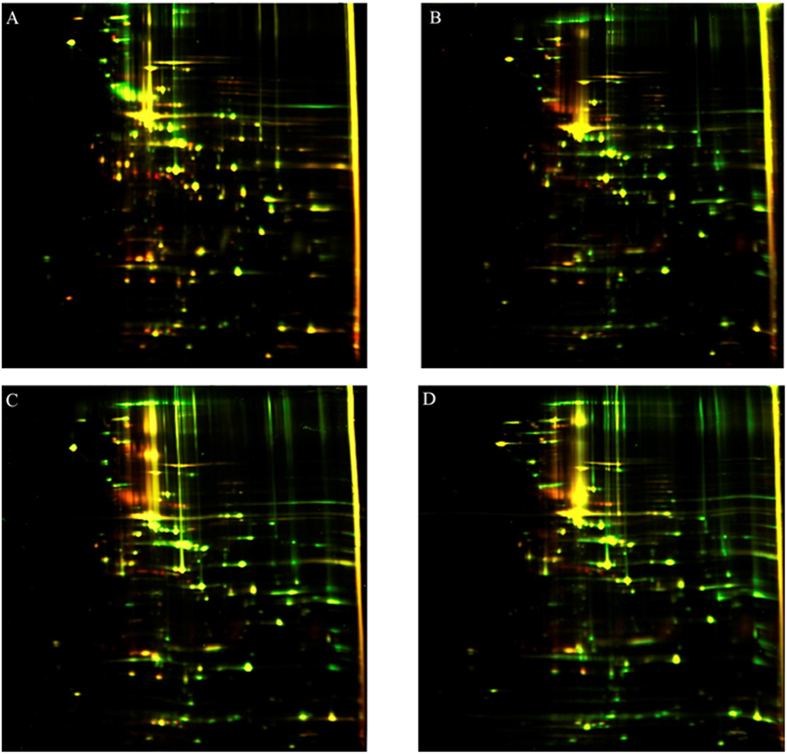
2D DIGE analysis of various *M. gallisepticum* isolated from HD3 cells after 7 weeks of infection (Cy3, green) and the laboratory strain *M. gallisepticum S6* (Cy5, red). (**A**) *M. gallisepticum* (green). (**B**) *M. gallisepticum* initially isolated from HD3 cells (green). (**C**) *M. gallisepticum* initially isolated from HeLa cells (green). (**D**) *M. gallisepticum* initially isolated from mES cells (green).

**Figure 5:**
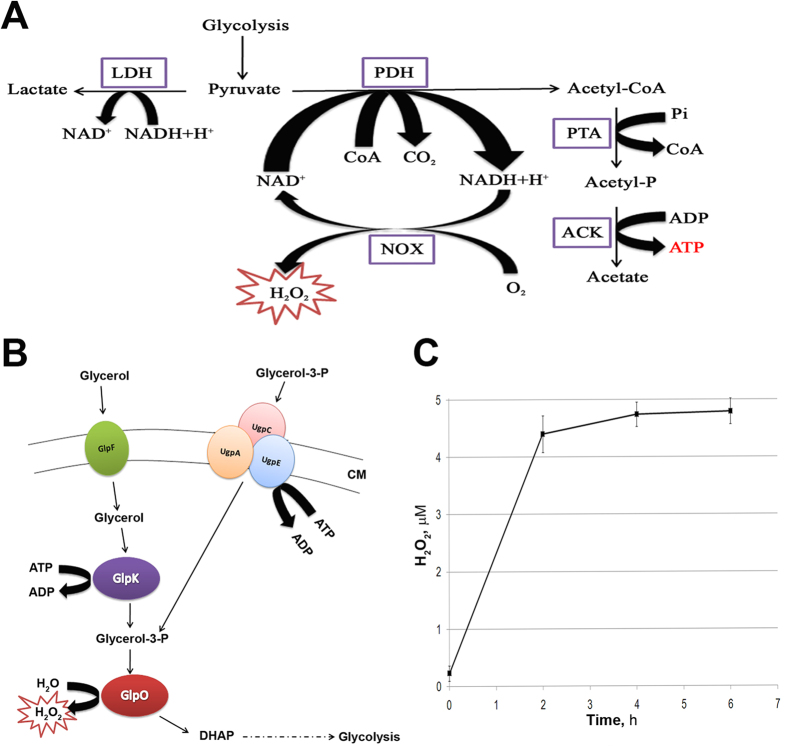
Production of hydrogen peroxide by *M. gallisepticum*. (**A**) Overview of pyruvate metabolism in *M. gallisepticum S6*. LDH-lactate dehydrogenase; PDH-pyruvate dehydrogenase complex; PTA–phosphotransacetylase; ACK-acetate kinase; NOX–NADH-oxidase. (**B**) Schematic illustration of the recovery pathway of glycerol and glycerol-3-phosphate in *Mycoplasma gallisepticum.* Free glycerol is transported into the bacterial cell by the membrane transporter GlpF. Then, the glycerol is phosphorylated by glycerol kinase GlpK, thereby forming a molecule of glycerol-3-phosphate. In addition, *M. gallisepticum* recycles free glycerol-3-phosphate through a transport system containing UgpACE ABC transporters. Further, glycerol-3-phosphate is converted to dihydroxyacetone phosphate by the oxidoreductase GlpO, secreting hydrogen peroxide. СМ–cell membrane; DHAP-dihydroxyacetone phosphate. (**C**) Determination of hydrogen peroxide release. Detection of H_2_O_2_ production during incubation of the eukaryotic cells HD3 with *M. gallisepticum* for 0, 2, 4 and 6 hours of co-cultivation using the Amplex Red Hydrogen Peroxide/Peroxidase Assay Kit (Thermo Fisher Scientific Inc, USA). Error bars indicate standard deviation (based on three independent experiments).

Chronic infection resulted in a similar pattern of protein changes regardless of the cell line used (Fig. 4, Supplementary Table S1). Proteins that were differentially expressed after 19 days of infection in HD3 cells (Fig. 3B) included several functional groups: oxidative stress defense, oxidoreductases, glycolysis, translation, gene expression regulation and proteins with unknown function. The most significant changes were observed for the proteins involved in redox potential homeostasis and oxidative stress defense (TrxA_1, TrxA_2, TrxB_1, OsmC, Dps). This set of proteins can reduce both organic and inorganic peroxides using NADH or NADPH. Dps is a multifunctional protein whose major function is DNA defense^24^. The oxidoreductases include 2,5-diketo-D-gluconic acid reductase, glyceraldehydes-3-phosphate dehydrogenase PutA and azoreductase AzoR. The latter is involved in the protecting of restored thiols pool in the cell. NADH oxidase is another oxidoreductase that is upregulated during infection. It utilizes reduced NADH using molecular oxygen as an electron acceptor. Its role is ambiguous because it produces reactive oxygen species (Fig. 5A). SpxA, which is upregulated after all types of infection, is a global regulator that acts on the level of transcription^25^. It modulates the affinity of RNA-polymerase for different promoters, resulting in the up- or downregulation of corresponding genes. Glycolytic proteins that are involved in the adaptation to the intracellular environment are pyruvate kinase, pyruvate dehydrogenase subunit B, triosephosphate isomerase, 6-phosphofructokinase, phosphoglycerate mutase and lactate dehydrogenase. Glyceraldehyde-3-phosphate dehydrogenase, which has been previously mentioned, is a glycolytic enzyme that catalyzes a bypass from glyceraldehyde-3-phosphate to glycerate-3-phosphate without 1,3-biphospho glycerate. Translation factors that respond to infection include EF-P and EF-Ts.

The trends of the oxidative stress response proteins and SpxA upregulation are more noticeable after 7 weeks of mycoplasma co-culture with HD3 cells (Fig. 3C, Supplementary Table S1). We also observed changes of chaperones DnaJ, trigger-factor-like proteins, the ABC-transporter OppA and several minor VlhA-hemagglutinins in addition to the major ones.

Importantly, only prolonged *in vitro* culture of the MIEC resulted in a return to the initial protein profile (Supplementary Figs S4 and S5). The mycoplasma was assayed after third, sixth, twelfth and sixteenth passages. Interestingly, mycoplasma that were isolated from three different cell lines and then re-cultured in HD3 cells for 7 weeks (a chronic infection model) had identical protein profiles as shown by 2D-electrophoresis (Fig. 4). Thus, we propose that *M. gallisepticum* may synchronize its metabolism with the cell after long-term persistence, which results in the similar response of the proteome.

### SpxA is a global regulator of the adaptation of *M. gallisepticum* to the intracellular medium

We constructed *M. gallisepticum* transformants that overexpressed *spxA* (Fig. 2E) and compared them to the control *M. gallisepticum S6* strain by 2D-electrophoresis (Fig. 2D). SpxA overexpression in *M. gallisepticum* increased the level of proteins with oxidoreductase activity and those responsible for protection against oxidative stress, such as OsmC, DkgA, Dps, TrxA_1, TrxB_1, flavodoxin, AzoR, and MsrA. Upregulation of the same proteins was observed in the MIEC, but in this case, we did not observe upregulation of NADH-oxidase. We observed a number of minor protein changes in *spxA*-overexpressing *M. gallisepticum* that were detected in the MIEC as well (Fig. 2D).

### MRM analysis demonstrates switching of hemagglutinin pattern of *M. gallisepticum* during infection

To study the suite of VlhA-hemagglutinins, we used an MRM-MS analysis of *M. gallisepticum* under previously described conditions. We used this method to determine VlhA-antigens because most of them are minor proteins that could not be detected by 2D-electrophoresis. Totally we analyzed representation of 33 VlhA-hemagglutinins. The VlhA profile of *M. gallisepticum* changes during an acute 24-hour infection (Supplementary Table S2). Furthermore, the direction of the change for half of the identified VlhA-antigens (GCW_02615, GCW_01930, GCW_01170, GCW_01930, GCW_91181, GCW_01160, GCW_01145, GCW_01195, GCW_01920, GCW_01940 and GCW_02390) is the same in all three cell lines. An abundance of a major VlhA GCW_01940 grew significantly in all three cell lines and a second, namely VlhA GCW_03350, demonstrated only a limited change. Among all of the cell lines used, mES cells evidenced the most notable changes in the representation of the VlhA-antigens, possibly because the invasion of the mycoplasma into embryonic stem cells is more difficult and proceeds by additional mechanisms.

An analysis of the variability of the VlhA-antigens during chronic infection revealed a number of interesting features: in all samples, we observed an increase in the VlhA-antigen GCW_01195 as well as a decrease in GCW_03350 (Supplementary Table S3). The latter represents one of two major antigens in the laboratory strain. Changes in GCW_01195 were observed during acute infection as well. We could conclude that VlhA GCW_01195 is a major hemagglutinin that responds most notably to intracellular localization. We found that the same antigens may behave differently during a first and second infection. The Vlh-antigens GCW_01160 and GCW_03335 demonstrated a significant increase during the first infection and did not change during the second. On the contrary, the expression of GCW_01155 and GCW_02440 changed only after the second infection.

### At the genomic level, single nucleotide polymorphisms (SNP) occurred in the MIEC VlhA-hemagglutinin genes

**Figure 6:**
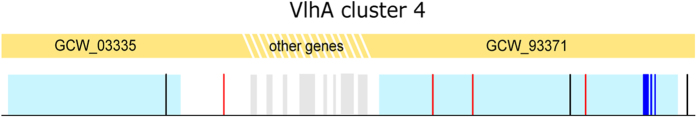
Distribution of SNPs localized in the *VlhA* cluster 4 genes in MIEC after acute (24 h) and chronic (7 weeks) infections. Lines indicate location of SNPs detected in *M. gallisepticum* isolated from HD3 cells. Black line-acute infection; blue line–chronic 7-week infection; red line–both acute and chronic 7-week infection.

We sequenced genomes of 10 different colonies of *M. gallisepticum* isolated from HD3 cells after acute infection and 10 colonies after chronic 7-week infection and compared to 12 reference mycoplasma colonies. We found a set of SNPs that were present only in the MIEC (Supplementary Table S4). These SNPs were predominantly localized in the VlhA-hemagglutinin genes. Part of these SNPs was localized in the intergenic regions. With increasing time of infection the number of SNPs found in the *vlhA* genes increased significantly. Most of the identified SNPs are localized in *vlhA* cluster 4 genes (Fig. 6) and we also observed changes in this cluster at the protein level (Supplementary Tables S2 and S3).

### *M. gallisepticum* undergoes metabolic reorganization during infection

Glycerol is a major nutrient for *M. gallisepticum* during infection. Therefore, we analyzed the glycerol utilization pathway using a metabolomic assay. The control cells and the MIEC were grown on a medium containing glycerol instead of glucose. We demonstrated an increase in dihydroxyacetone phosphate (DHAP) in the MIEC (Supplementary Fig. S6, Supplementary Table S5), which indicated that mycoplasma cells actively used glycerol and glycerol-containing molecules as a substrate during intracellular localization. Simultaneously, *M. gallisepticum* contain a pathway for converting glycerol-3-phosphate into DHAP with the concomitant formation of hydrogen peroxide (Fig. 5B). It has been demonstrated that the hydrogen peroxide produced by mycoplasma during glycerol utilization may serve as a pathogenicity factor^26^.

To confirm the use of glycerol as a substrate by mycoplasma cells during infection, the peroxide concentration was determined at 0, 2, 4 and 6 hours of co-incubation with eukaryotic cells. The results are shown in Fig. 5C. The hydrogen peroxide concentration depends directly on the time of incubation of *M. gallisepticum* with the eukaryotic cells, and the hydrogen peroxide concentration ultimately reaches a plateau.

**Figure 7:**
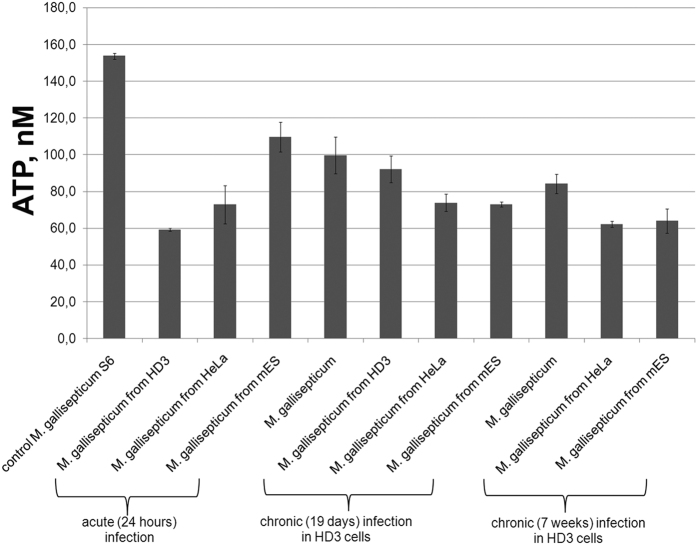
Measurement of the ATP concentration. Detection of ATP production in MIEC after acute and chronic infections and control *M. gallisepticum S6*. The data represent the mean (±SD) of three independent experiments performed in duplicate.

Metabolic pathways of mycoplasmas are mostly associated with energy generation, while the biosynthetic capacity is greatly reduced. We measured the intracellular ATP concentration to characterize the total metabolic outcome of MIEC in comparison with the control strain (Fig. 7). The ATP concentration was measured using a luciferase-based firefly system. We detected a significant decrease in the amount of ATP in MIEC compared to the control mycoplasma strain.

## Discussion

In this study, we observed a phase transition of *M. gallisepticum* in response to interaction with a various host cells. *M. gallisepticum* switches to one stable alternate phase state after invasion of three different eukaryotic cells and the relaxation of this state occurs after several passages in culture medium. Interestingly, the response did not depend on the type of eukaryotic cell infected. This phase transition was manifested on the proteomic, metabolomic and genomic levels.

The state of the cell can be likened to a point in a multi-dimensional phase space, where the coordinates are concentrations of all molecules within the cell. In these terms, the point position can be designated as the phase state. The state of the cell under normal conditions should oscillate in the vicinity of an attractor. An external stimulus such as a stress drives the cell state away from the attractor. However, this new state is transient: it is maintained solely by extracellular conditions. When external conditions are returned to the initial ones, the cell rapidly returns to its initial state^27^. This is achieved by regulatory systems that force both processes–stress response and recovery. An opposite phenomenon is known as bistability^28^,^29^, which may occur even in the absence of an external stimulus and result in at least two stable attractors. Switch to one results in a long-term persistence around it.

In our work we observed the transition of *M. gallisepticum* to a new stable attractor as a result of host-pathogen interactions. One passage in culture resulted in 10-fold growth of the cellular biomass and a respective dilution of initially existing proteins (including regulators). The average protein upregulation in our study was 2-4-fold, which would be nullified after one passage. In our model, the cells retained the altered state for the third passage and gradually returned to the initial state after sixteenth passage (Supplementary Fig. S4). During a longer infection time more changes persisted up to the sixteenth passage (Supplementary Fig. S5). Thus, the stability of this state cannot be due only to the accumulation and persistence of the proteins that were synthesized under intracellular conditions. The cellular phase state around this new attractor is significantly stable. The return to the initial state cannot be accomplished solely by the regulatory systems that have driven the cell to the new state or even by the dilution of the existing proteins.

It is possible that a similar phenomenon takes place when primary isolates are transforming into cell culture. One can speculate that the stability of a non-optimal (under artificial conditions) proteome composition does not allow the regulatory system to adapt cellular metabolism to the new conditions. As a result, many isolates ultimately cease growing. The lower cellular energization (i.e., the ATP level) that was observed after infection may be of the same origin because we worked with re-cultured cells. Probably the optimal proteome present under intracellular conditions is suboptimal when the cells are placed in a culture medium.

Furthermore, in this study, we demonstrated for the first time the ability of *M. gallisepticum* to infect murine mES and chicken HD3 cells in culture. According to most of the published data, *M. gallisepticum* is considered to be an avian parietal parasite, but recently, an increasing amount of data have demonstrated its ability to invade eukaryotic cells and thus disseminate through the body of the host^8^,^9^,^11^. The infection of HeLa cells has been previously demonstrated^8^. We used eukaryotic cell lines with different properties to study interactions with different hosts. HD3 cells were the closest model to *in vivo* infection. It has been shown that *M. gallisepticum* could invade chicken erythrocytes and systemically spread throughout the host^9^. Murine embryonic stem cells provide an alternative cell-based system to terminally differentiated cells for infection studies^30^.

We used proteomic rather than transcriptomics technologies because recent studies indicate that the mRNA level may have a poor correlation with the protein level. It was observed in our previous work for *M. gallisepticum* in stress^13^. Similar observations were made for different bacteria including *E. coli*^31^, *Desulfovibrio vulgaris*^32^, and *Lactococcus lactis*^33^. We propose that changes on the proteomic level reflect functional changes to a greater extent.

The intracellular environment induced a global reorganization of the *M. gallisepticum* proteome. The impact of different cell cultures on *M. gallisepticum* was similar and the similarity increased with the duration of infection (Figs 3 and 4). We propose that the observed upregulation of the glycolytic enzymes is a response to the increased demand for energy during infection. Glycolytic proteins and translation elongation factors may be involved in secondary adhesion as well^34^. It is possible that the primary attachment of *M. gallisepticum* to the cell membrane induces auxiliary proteins that enhance adhesion.

One more mechanism of adhesion and adaptation of the *M. gallisepticum* to the host is change of surface VlhA-antigens. We have found that the infection of eukaryotic cells promotes changes in the suite of VlhA-antigens in *M. gallisepticum* along with the upregulation of defense proteins. The number of varying VlhA-antigens is much less during a chronic infection (Supplementary Table S3), compared with an acute infection (Supplementary Table S2). We can assume that during the initial contact of *M. gallisepticum* with the host cell and for further persistence, it needs a more diverse and a more highly expressed repertoire of VlhA-antigens. This idea is in accordance with previous findings^7^,^35^,^36^. Interestingly, in most cases, these changes are similar for different eukaryotic cells. In the same time, each cell line promotes specific variations of VlhA. Thus, a pool of VlhA-antigens exists that primarily responds upon initial contact with a host cell. After the initial infection, *M. gallisepticum* seems to retain some degree of adaptation to the intracellular environment, so it does not need a major reorganization of VlhA as it did for the first time.

Analysis of different MIEC genomes identified SNPs mostly in the *vlhA* genes (Supplementary Table S4). Perhaps these SNPs are associated with the reversible loss or acquisition of surface components resulting in different exposed antigens, which is the primary strategy for survival among bacterial pathogens^10^,^37^. Most SNPs detected in the coding region of *vlhA* (not in the intergenic region) were localized within genes whose respective proteins change in abundance during infection, which could be a possible mechanism of additional antigenic variability of VlhA to evade the immune system of the host. Genomic rearrangements were found by an analysis of *M. gallisepticum* isolated from house finches that have not been considered as potential hosts^6^, in agreement with our data. Thus, it can be assumed that the variability of the genome is another mechanism of adaptation to the host.

Oxidative stress protection and carbohydrate metabolism were the functional classes of proteins that were most affected during infection. We found that the abundance of SpxA protein increased in both acute and chronic infection in all cases (Fig. 3, Supplementary Table S1). This data are in agreement with *Listeria monocytogenes* study where the gene similar to the *B. subtilis spx* gene was upregulated during intracellular growth, suggesting that Spx might play a role in invasion and intracellular survival^38^. SpxA is a highly conserved regulatory protein in low-GC-content Gram-positive bacteria^39^. SpxA binds with the C-terminal domain of the α-subunit of RNA polymerase and modulates its affinity to different promoters. In *B. subtilis,* SpxA is upregulated in response to various stress conditions, including heat, salt and superoxide stress^40^. Nakano *et al.* showed that this protein activates the transcription of *trxA* (thioredoxin) and *trxB* (thioredoxinreductase) in response to oxidative stress^41^. A similar function of SpxA was shown in *Bacillus anthracis* and *Enterococcus faecalis*^42^,^43^. SpxA is activated via the oxidation of a CXXC motif to a disulfide, which senses the intracellular redox potential^44^. SpxA homologs may be involved in numerous functions including virulence, stress response and competence development^42^,^43^,^45^,^46^,^47^. In this study, we demonstrated that besides the upregulation of the TrxA and TrxB proteins, SpxA induces the upregulation of a set of oxidoreductases and several other proteins of glycolysis, translation and cytadherence (Fig. 2D). A similar response was observed during intracellular infection (Fig. 2A–C). Moreover, in our previous mycoplasma heat shock study we did not observe upregulation of SpxA protein^13^. On this basis, we make the assumption that the SpxA protein plays an important role in the regulation of the adaptation of *M. gallisepticum* to intracellular conditions.

However, why does *M. gallisepticum* activate a system to regulate and protect against oxidative stress during infection? We found that NADH-oxidase was upregulated in *M. gallisepticum* during infection. NADH-oxidase utilizes molecular oxygen as an electron acceptor and produces hydrogen peroxide as a side-product. *M. gallisepticum* contains two pathways for pyruvate metabolism. The first is via lactate dehydrogenase, and the second is via pyruvate dehydrogenase. The first is balanced for the production and utilization of NADH. The second allows the production of an additional molecule of ATP, but in this case, the production of NADH does not equal its utilization. Thus, NADH-oxidase may serve to regenerate NAD and allow the pathway to continue. Upregulation of NADH-oxidase may be a mean to accelerate ATP synthesis via activation of a second pathway to cover increased demand of energy during infection (Fig. 5A). The intracellular ATP concentration decreases after infection (Fig. 7), which may indicate increased ATP consumption. The observed upregulation of the glycolytic enzymes supports this idea (Fig. 3, Supplementary Table S1).

As we demonstrated, *M. gallisepticum* shows an increased use of glycerol or glycerophosphodiesters as an energy source during infection, which results in the accumulation of DHAP (Supplementary Fig. S6). This pathway is another source of hydrogen peroxide (Fig. 5B). Activation of either utilization of pyruvate to acetate or glycerol to pyruvate in *M. gallisepticum* in inevitably results in an increased production of endogenous hydrogen peroxide against which the bacteria have to mount defenses. The observation of endogenous hydrogen peroxide production supports our hypothesis (Fig. 5C).

Apart of the toxic side-products hydrogen peroxide may play a role as a pathogenicity factor. It may initiate lipid peroxidation in the host cell membrane, thereby compromising the integrity and permeability of the eukaryotic cell membrane, facilitating the entry of the bacteria. The role of hydrogen peroxide in pathogenicity may be ambiguous. On the one hand, hydrogen peroxide formed via glycerol-3-phosphate utilization is a major virulence factor of *Mycoplasma pneumonia*^26^, which supports the idea of the importance of the intensification of metabolism. On the other hand, recent findings in *M. gallisepticum* show that the genes for glycerol metabolism do not seem to be important for pathogenicity in the natural host^48^. Therefore, the reason that *M. gallisepticum* synthesizes hydrogen peroxide during intracellular infection is of great interest. We hypothesize that hydrogen peroxide is required for mycoplasma to change the surface VlhA-antigens acting either as a signaling molecule itself or through lipid peroxidation of the own membrane. In conclusion, we suggest several possible roles for hydrogen peroxide in infection: (i) a cytotoxic effect as a pathogenicity factor, (ii) a signaling molecule that activates processes required for adaptation during adhesion and intracellular invasion, (iii) the promotion of the invasion of the host cell by *M. gallisepticum*, (iv) activation of the regulatory protein SpxA, and (v) simply as a by-product of NADH-oxidase reactions that produce ATP in response to an increased demand for energy during infection.

Assumedly switching mechanism to another phase state has evolved as an adaptation to various external conditions for the best arrangement and survival of bacteria. In the future, it will help the more detailed study of the interaction mechanisms of the bacteria with the host and thus shed light on bacterial evolution.

## Materials and Methods

### Mycoplasma infection and gentamicin invasion assay

*M. gallisepticum S6* cells were cultivated and passaged as described previously^49^. Cell lines Human cervical cancer cells HeLa-229 (ATCC CCL-2) and murine embryonic stem (mES) (ATCC SCRC-1011) were obtained from the American Type Culture Collection (ATCC; Manassas, USA) and certified to be free of mycoplasmas. HeLa were cultured as described in ref. 50. mES cell were cultured in DMEM containing 15% heat-inactivated FBS, Penicillin-Streptomycin (100 U/mL), 2 mM L-glutamine, 1xNEAA, 10 ng/ml mrLIF in 0,1% gelatin-coated Petri dishes. Chicken erythroblast cell line HD3 (clone A6 of line LSCC^51^,^52^) was kind gift from professor S. V. Razin (Institute of Gene Biology, Russian Academy of Sciences) and was cultivated as described previously^53^. Cell cultures were regularly checked for mycoplasma contamination by PCR. The gentamicin invasion assay was carried out as previously described^8^. We used the concentration of gentamicin 600 μg/ml. The survival of mycoplasmas after gentamicin treatment was estimated using the colored indicator BromPhenolRed. Cell lines were infected with the bacterium *Mycoplasma gallisepticum S6* in a ratio of 1:1,000 respectively and cultured for 24 hours in a CO_2_-incubator. For the model of chronic infection HD3 cells were cultured with mycoplasma for 19 days and 7 weeks. After the appropriate cultivation time with mycoplasma eukaryotic cells were treated with gentamicin. Culture of *M. gallisepticum* was taken in late-log phase. For the control mycoplasma cultivated in semiliquid and liquid medium as a MIEC was taken. Invasion frequency was calculated as percentage ratio of CFU of intracellular mycoplasmas to CFU of mycoplasmas added initially. All the above experiments were done in duplicates and performed at least thrice under the same conditions. See Supplementary Information for details.

### Fluorescent labeling of eukaryotic cells with *M. gallisepticum* and confocal microscopy

The presence of mycoplasmas within eukaryotic cells was investigated by laser scanning confocal microscopy. F-actin in eukaryotic cells was stained with Alexa Fluor 568 phalloidin, and the nuclei of eukaryotic cells and mycoplasma were stained with DAPI. Samples were examined with an LSM 510 Meta confocal laser scanning microscope (Carl Zeiss MicroImaging GmbH, Jena, Germany). See Supplementary Information for details.

### Two-dimensional difference gel electrophoresis (2DE)

The 2 DE, samples trypsin digestion with subsequent MALDI analysis were performed as previously described^54^.

### SpxA overexpression in *M. gallisepticum*

The ORF of the *spxA* gene was amplified from *M. gallisepticum* genomic DNA and cloned into a pRM5 transposon-based vector between the XhoI and NcoI sites (Fig. 2E), which is a variant of a previously designed vector^13^. See Supplementary Information for details.

### Trypsin digestion in solution

Сells were washed three times with PBS, pH 7.5. Cell pellet was treated with 3 μl of 10% RapiGest SF (Waters) and 1 μl nuclease mix for 30 min at 4 °C, then resuspended in 37 μl of 100 mM NH_4_HCO_3_, vortexed and heated at 100 °C for 5 min. After cooling to room temperature cell debris was removed by centrifugation at 15,000 g for 5 min. Protein cysteine bonds were reduced with 10 mM DTT in 5 mM NH_4_HCO_3_ for 30 min at 60 °C and alkylated with 30 mM iodoacetamide in the dark at RT for 30 min. The step with adding DTT was repeated. Clarified extract protein concentration was estimated using Bradford Protein Assay Kit (BioRad). Trypsin (Trypsin Gold, Mass Spectrometry Grade, Promega) was added in 1/50 w/w trypsin/protein ratio and incubated at 37 °C overnight. To stop trypsinolysis and degrade the acid-labile RapiGest surfactant, trifluoroacetic acid (TFA) was added to the final concentration of 0,5% v/v (the pH should be less than 2.0), incubated at 37 °C for 45 min and the samples were centrifugated at 15,000 g for 10 min to remove the surfactant. Hydrolyzate was desalted using a Discovery DSC-18 Tube (Supelco) according to the manufacturer protocol. Peptides were eluted with 700 μL 75% ACN, 0.1% TFA, dried in a SpeedVac (Labconco) and resuspended in 3% ACN, 0.1% TFA to the final concentration of 5 μg/μL.

### IDA and MRM LC-MS/MS analysis

Analysis was performed as previously dicribed^55^. For protein identification, wiff data files were analyzed with ProteinPilot 4.5 revision 1656 (Sciex, USA) using search algorithm Paragon 4.5.0.0 revision 1654 (Sciex, USA) and a standard set of identification settings to search against *M. gallisepticum S6* CP006916.2NCBI protein database supplied with common contaminants. The following parameters were used: alkylation of cysteine-iodoacetamide, trypsin digestion, TripleTOF 5600 equipment, species: none, thorough search with additional statistical FDR analysis. Peptide identifications were processed with default settings by a ProteinPilot software built-in ProGroup algorithm. The final protein identification list was obtained with the threshold reliable protein ID unused score calculated by ProteomicS Performance Evaluation Pipeline Software (PSPEP) algorithm for 1% global FDR from fit.

Quantitative LC-MS protein analysis was performed on the basis of MRM methodology on QTRAP 4500 (Sciex, USA) triple quadrupole mass spectrometer equipped with a NanoSpray III ion source (Sciex, USA) coupled to an expertNanoLC400nano-HPLC system (Eksigent, USA). The details of the MRM analysis are described in the Supplementary Information.

### Metabolomic assay

For metabolite analysis *M. gallisepticum* (25 ml) was grown in a liquid medium, 1 ml of liquid culture was used for protein quantification by Bradford assay and following normalization of metabolomic results. Metabolite extraction was developed on the basis of a previously reported cold methanol extraction protocol^56^. In addition 10 μl of isotope labeled 1 mg/ml L-Glutamic acid-^13^C_5_ was added to metabolites mix as internal standard (IS). Mass-spectrometry metabolite analysis was performed in MRM mode with following parameters. Positive and negative ionization modes: dwell time of each transition acquisition was 10 msec, maximum loop time was 2.245 msec, 5 and 11 time segments were used respectively (Table S6). Resolution of Q1 and Q3 were unit for both acquisition modes. Time segments of MRM analysis, collision energy and retention time of different metabolites was selected based on HPLC-MS/MS analysis of chemical standards.

Chromatography separation was carried out using HPLC 8030 Shimadzu system (Kyoto, Japan) and analytical column Zorbax RX-SIL NB 150 mm × 2.1 mm × 5 μm from Agilent Technologies (USA) supplemented with Zorbax RX-SIL 4-Pack analytical guard column 4.6 mm × 12.5 mm × 5 μm (Agilent Technologies).

Chromatographic analysis was performed with the following parameters: auto-sampling temperature, 20 °C; analytical column temperature, 32 °C; injection volume, 10 μl; solvent flow rate, 500 μl/min. The following solvents were used as eluting solutions: eluent A was 20 mM ammonium acetate/0.25 mM ammonium hydroxide in water/acetonitrile mixture of 95:5 ratio, pH 8.02; eluent B was pure acetonitrile. The gradient of the solvent transition was as follows: for positive and negative ionization mode *t = *0, 100% B; *t* = 15 min, 0% B; *t* = 18 min, 0% B; *t* = 19 min, 100% B; *t* = 32 min, 100% B.

The instrument control and the data processing were done by workstation “LabSolutions LCMS” Version 5.75 (Shimadzu Corporation, Kyoto, Japan). Metabolites were analyzed in the MRM mode using scheduled windows with two transitions per compound. [M + H]^+^ served as precursor ion, and the one transition was used (Table S6) to the quantify difference in metabolites concentration. Identification of metabolites was based on the retention time accordance with a tolerance of ±0.5 min; the presence of two identification points (precursor ion and one fragment ion). Quantification was based on MRM peak area normalized on the IS peak area and total protein content in each sample. We used following peak integration parameters: Width 30 sec, Slope 1000/min, Min Area/Height 10 counts, Signal/Noise detection limit 3.

### Genome sequencing and analysis

Genomic DNA from individual cultures was isolated as previously described^49^. The DNA (100 ng for each sample) was disrupted into 200–300 bp fragments using the Covaris S220 System (Covaris, Woburn, Massachusetts, USA). Barcode shotgun libraries were prepared by the Ion Xpress™Plus Fragment Library Kit (Life Technologies). PCR emulsion was performed by the Ion PGM™Template OT2 200 Kit (Life Technologies). DNA sequencing was performed by the Ion Torrent PGM (Life Technologies) with the Ion 318 chip v2 and the Ion PGM™Sequencing 200 Kit v2 (Life Technologies).

For the detection of single nucleotide variants relative to the reference, a reference-based mapping approach via bowtie2^57^ and samtools mpileup^58^ tools were used. On average 93% of reads mapped to the reference genome. We skipped alignments with mapping quality (mapQ) less than 10. Variants were called using the samtools mpileup command with options -C50 -D -S. Variants were filtered using the following criteria: (1) the depth of high-quality coverage larger than 60, (2) at least 75% of reads at the site supporting the call, (3) a homozygous call under a diploid model. We identified SNPs by comparing calls between the control genomes and the MIEC genomes.

### Determination of hydrogen peroxide production

The hydrogen peroxide production in *M. gallisepticum* during intracellular localization was determined using the Amplex^®^ Red Hydrogen Peroxide/Peroxidase Assay Kit (Thermo Fisher Scientific Inc, USA) according to standard protocol with some modifications. See Supplementary Information for details.

### Measurement of ATP concentration in *M. gallisepticum* cells

Detection of ATP was performed as previously described^59^. Also see Supplementary Information for details.

### Statistical analysis

Invasion frequencies, the determination of hydrogen peroxide and ATP production are expressed as the mean ± standard deviation of *n* independent values. The significance of differences between experimental means was determined with Student’s *t* test. Differences with P < 0.05 were considered significant.

## Additional Information

**How to cite this article**: Matyushkina, D. *et al.* Phase Transition of the Bacterium upon Invasion of a Host Cell as a Mechanism of Adaptation: a *Mycoplasma gallisepticum* Model. *Sci. Rep.* **6**, 35959; doi: 10.1038/srep35959 (2016).

## Supplementary Material

Supplementary Information

Supplementary Table S1
