# Supplementary material for: Phase Transition of the Bacterium upon Invasion of a Host Cell as a Mechanism of Adaptation: a Mycoplasma gallisepticum Model
Source: Sci Rep. 2016 Oct 24;6:35959. doi: 10.1038/srep35959 (PMC5075909; doi:10.1038/srep35959)
Supplement: Supplementary Information [file srep35959-s1.pdf]

Phase Transition of the Bacterium upon Invasion of a Host Cell as a Mechanism of Adaptation: a *Mycoplasma gallisepticum* Model

Daria Matyushkina, Olga Pobeguts, Ivan Butenko, Anna Vanyushkina, Nicolay Anikanov, Olga Bukato, Daria Evsyutina, Alexandra Bogomazova, Maria Lagarkova, Tatiana Semashko, Irina Garanina, Vladislav Babenko, Maria Vakhitova, Valentina Ladygina, Gleb Fisunov, Vadim Govorun

d.matyushkina@gmail.com

Supplementary Methods

Gentamicin invasion assay

Cell lines were infected with the bacterium *Mycoplasma gallisepticum S6* in a ratio of 1:1,000 respectively and cultured for 24 hours in a CO_2-incubator. For the model of chronic infection HD3 cells were cultured with mycoplasma for 19 days and 7 weeks. Since mES and HeLa cell lines are adhesive, after 24h incubation of mES and HeLa with *M. gallisepticum* cells were washed from unbound mycoplasma 3 times with PBS buffer. Thereafter, extracellular bacteria were killed by incubation in medium containing 600 µg / ml of gentamicin and incubated for 3 hours at 37 °C, 5% CO_2. A higher concentration of 600 µg / ml was used to ensure the reliability of the assay and was experimentally determined to be sufficient to kill 100% of *M. gallisepticum* in 3 h duration. After gentamicin treatment, the cells were washed two times with PBS. The final pellet was resuspended in preheated medium for *M. gallisepticum* and plated on semi-solid medium containing 0.3% bacteriological agar (Sigma-Aldrich). Cell suspension with cultural media for *M. gallisepticum* was used as a negative control. Invasion frequency was calculated as percentage ratio of CFU of intracellular mycoplasmas to CFU of mycoplasmas added initially. Then colonies of *M. gallisepticum* were subcultured in liquid medium and cultured

until the logarithmic growth phase. The cell pellet was obtained by centrifugation at 8,000g at 4 °C, 10 min.

Fluorescent labeling of eukaryotic cells with *M. gallisepticum* and confocal microscopy

Eukaryotic cells were washed and infected with *M. gallisepticum* cultures by using a procedure described above for the gentamicin invasion assay. The infected cells were gently washed two times with PBS, fixed in 4% paraformaldehyde for 15 minutes at room temperature and rinsed two times with PBS. Fixed cells were permeabilized with a solution of 0.1% Triton X-100 in PBS for 3 min and washed two times with PBS. 5 µl methanolic stock solution of Alexa Fluor 568 phalloidin (Invitrogen) were diluted in 200 µl solution of 1% BSA in PBS. F-actin in eukaryotic cells was stained with phalloidin solution for 20 minutes at room temperature and washed two times with PBS. Nuclei of eukaryotic cells and mycoplasma were stained with DAPI solution (Invitrogen) (0.5 µg/ml in PBS) for 3 minutes at room temperature and rinsed two times with PBS, air-dried and mounted with a 24 × 50 mm coverslip and Bio Mount (Bio-Optica, Italy).

Samples were examined with LSM 510 Meta confocal laser scanning microscope (Carl Zeiss MicroImaging GmbH, Jena, Germany) using x63 oil objective (NA 1.40). Diode (405 nm) and helium-neon (543 nm) lasers were used for specific excitation of the fluorescent dyes DAPI and Alexa Fluor 568, respectively. The Z-stacks of fluorescent images were collected at 0.37-0.38 µm interval.

MRM LC-MS/MS analysis

To obtain transition list for proteins of interest, peak lists with protein identification (.group files) obtained for IDA experiments were loaded into Skyline software, where no more than 10 unique peptides per protein with global FDR rate from fit according to PSPEP were selected with additional constrains on peptide length (7-20 amino acid residues), cleavage (fully tryptic peptides without missed cleavage sites or potential ragged ends) and modifications (only carbamidomethylation of cysteins allowed). For each precursor of charge 2, 3 or 4, 3 or 4 most intense fragment ions were selected. Raw data was obtained by duplicate injection of each sample with the same chromatographic setup with total cycle time of 3.3 s. After each sample correction for retention time drift was applied, scheduled MRM time window for each precursor comprised 5 minutes.

Processing of data included peak selection (with manual review), peak integration and export utilizing Skyline software. Further analysis was performed by homemade script in R. The algorithm included scaling normalization of LC-MS replicates on peptide level and of each set of samples on protein level (based on 2 most intense peptides). Proteins with mean change across 3 biological replicates was above the threshold of 1.2 were reported as significant.

SpxA overexpression in *M. gallisepticum*

The transformation of *M. gallisepticum* was performed by electroporation as described previously[1]. Transformants were selected on a semiliquid medium supplied with 2 μg/ml of tetracyclin. The transposon insertions were confirmed and mapped by PCR and

Sanger sequencing from the chromosome. The overexpression of the *spxA* gene was confirmed by real-time PCR.

Determination of hydrogen peroxide production

The hydrogen peroxide production in *M. gallisepticum* during intracellular localization was determined using the Amplex® Red Hydrogen Peroxide / Peroxidase Assay Kit (Thermo Fisher Scientific Inc, USA). 50µl of the standard curve samples, controls and suspension of eukaryotic cells and *M. gallisepticum* were pipeted into individual wells of a microplate. The standard curve samples were made in the mix of media for eukaryotic cells and media for *M.gallisepticum.* HD3 cells incubated with media for *M. gallisepticum* were used as a control samples. 50µl of the Amplex® Red reagent / HRP working solution was added to each microplate well and incubated for 1hour at room temperature in the dark. Fluorescence was measured with a fluorescence microplate reader (xMark Microplate Spectrophotometer, BioRad) using absorbance 560nm. Background fluorescence, determined for a no-H_2O_2 control reaction, has been subtracted from each value. Experiments were done in three independent experiments in triplicate.

Measurement of ATP concentration in *M. gallisepticum* cells

Cultures of *M. gallisepticum* control strain and MIEC were taken in late-log phase. Normalization to the cell number was performed using quantitative RT-PCR. To determine the extracellular ATP in the microcuvette were added 20 µl of sample and 100 µl ATP-reagent (Lyumtek, Russia). An ATP-bioluminescence integral signal was measured within 30 seconds (RLU/s) at luminometer LUM-1 (Lyumtek, Russia). The measurement was carried out in three independent experiments performed in duplicate. The $7.5 * 10^{-9}$ M ATP

control solution (Lyumtek, Russia) was prepared in mycoplasma culture medium. To determine the total ATP were taken 50 µl of the sample in microtube, added 450 µl of anhydrous dimethylsulfoxide and mixed thoroughly. After 1 min 20 µl of obtained extract were added to microcuvette and ATP levels were measured as described above. ATP concentration was calculated by the formula:

$$[ATP]_{sample} = 10 \cdot 7{,}5 \cdot 10^{-9} = 7{,}5 \cdot 10^{-8}((I_{sample})/ (I_{control})), \text{mol/l}$$

Coefficient "10" is the dilution factor of the sample in the preparation of the extract.

Normalization to the cell number was performed using quantitative RT-PCR.

References:

1. Mazin, P. V. *et al.* Transcriptome analysis reveals novel regulatory mechanisms in a genome-reduced bacterium. *Nucleic Acids Res.* **42,** 13254–13268 (2014).

Supplementary figures

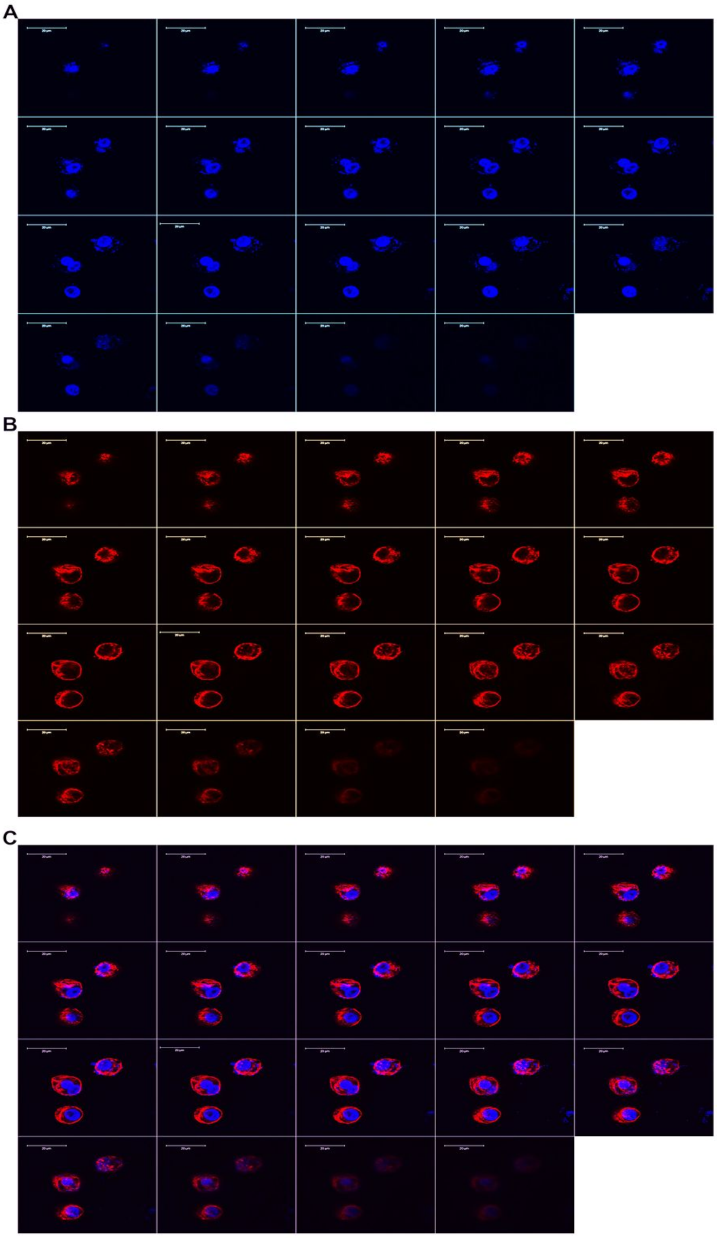

S1 Figure. Composite confocal image of 19 Z sections of HD3 cells incubated with *M. gallisepticum* for 24 hours.

0,37 - 0,38 μm steps. DAPI fluorescence showing cell nuclei and mycoplasmas stained blue (A), Alexa Fluor 568 phalloidin fluorescence showing eukaryotic cell F-actin stained red (B). The merged fluorescent image is shown in (C). Scale bars, 20 μm.

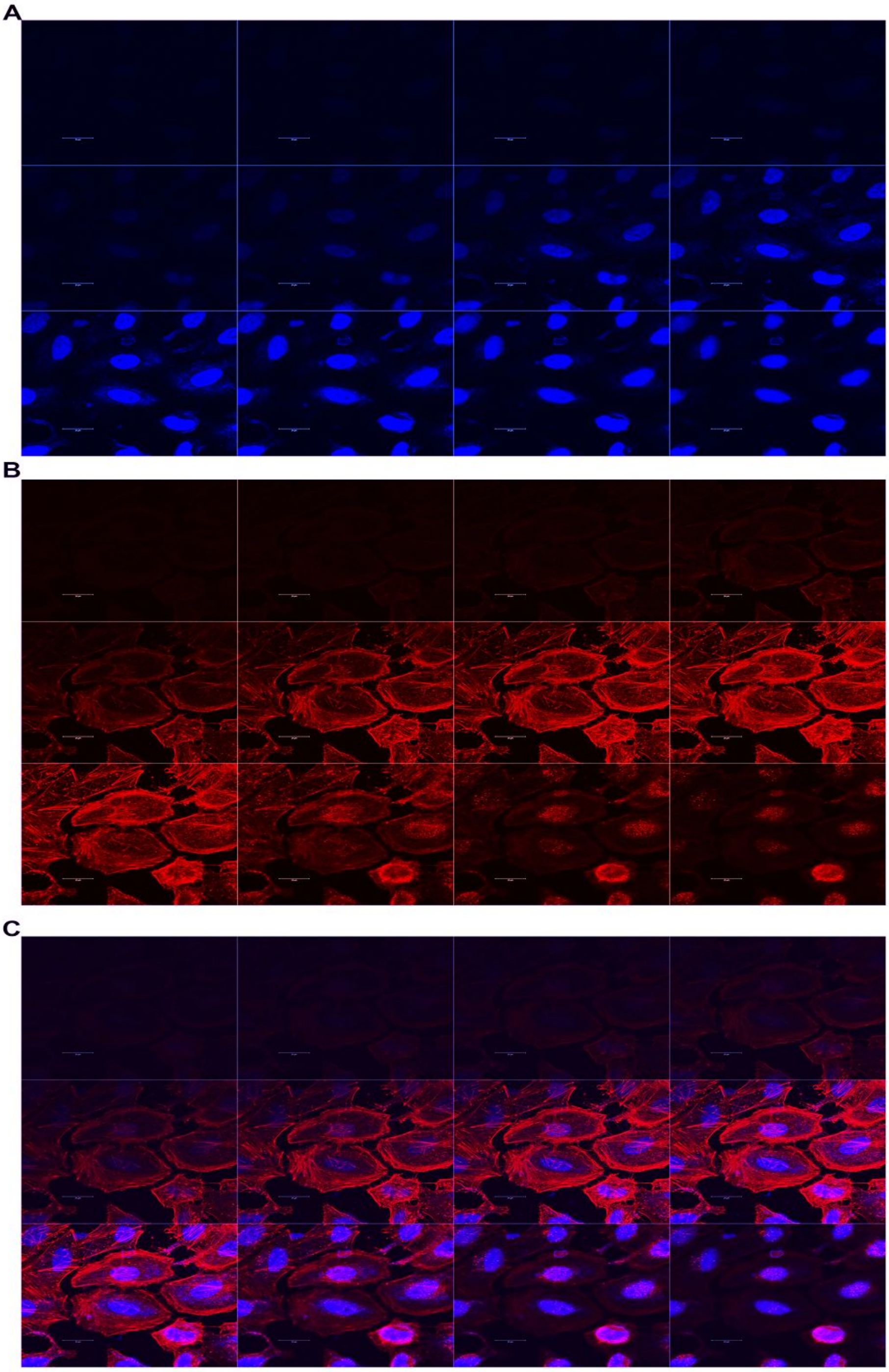

S2 Figure. Composite confocal image of 12 Z sections of HeLa-229 cells incubated with *M. gallisepticum* for 24 hours.

0,37 - 0,38 μm steps. DAPI fluorescence showing cell nuclei and mycoplasmas stained blue (A), Alexa Fluor 568 phalloidin fluorescence showing eukaryotic cell F-actin stained red (B). The merged fluorescent image is shown in (C). Scale bars, 20 μm.

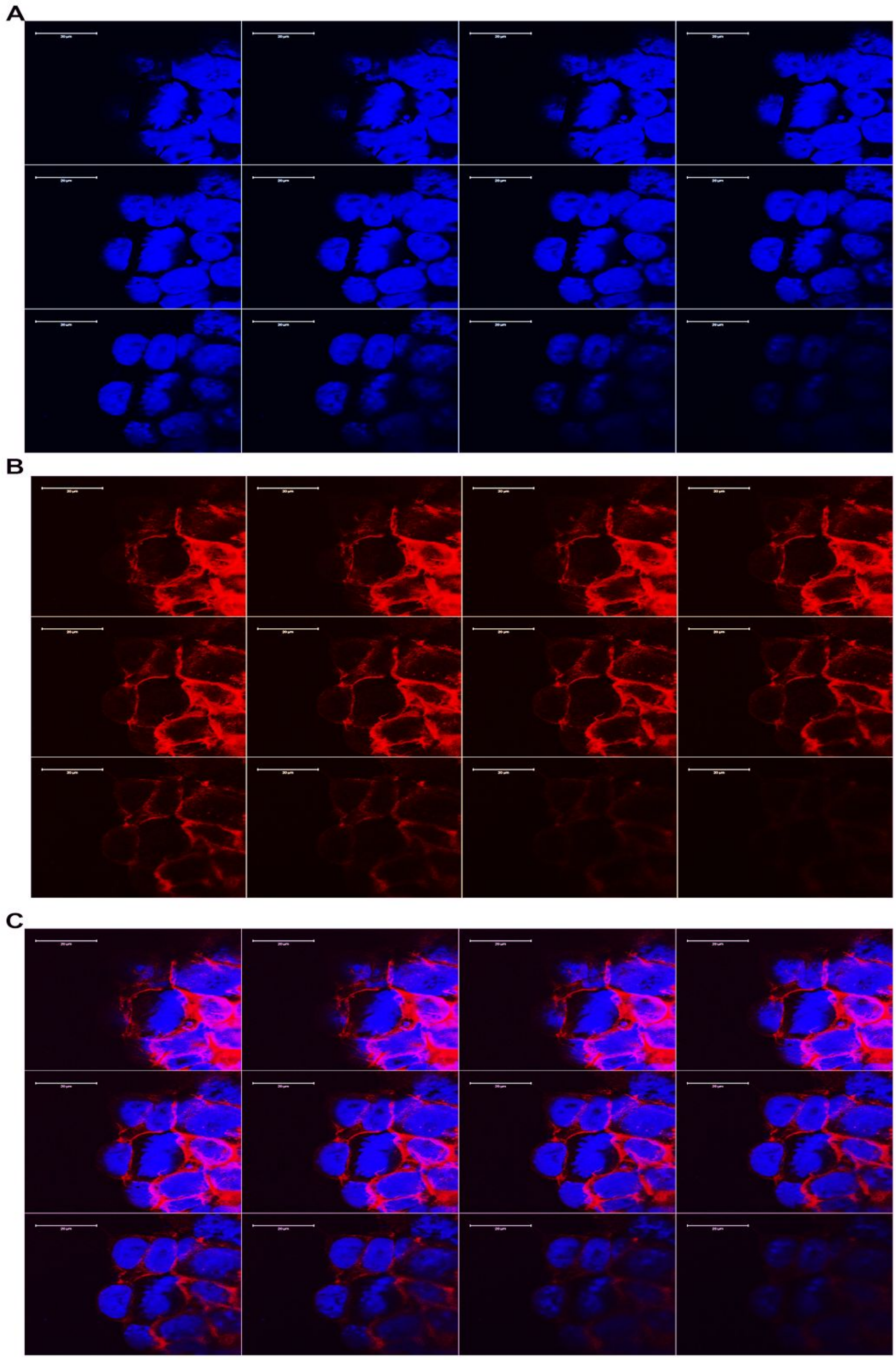

S3 Figure. Composite confocal image of 12 Z sections of mES cells incubated with *M. gallisepticum* for 24 hours.

0,37 - 0,38 µm steps. DAPI fluorescence showing cell nuclei and mycoplasmas stained blue (A), Alexa Fluor 568 phalloidin fluorescence showing eukaryotic cell F-actin stained red (B). The merged fluorescent image is shown in (C). Scale bars, 20 µm.

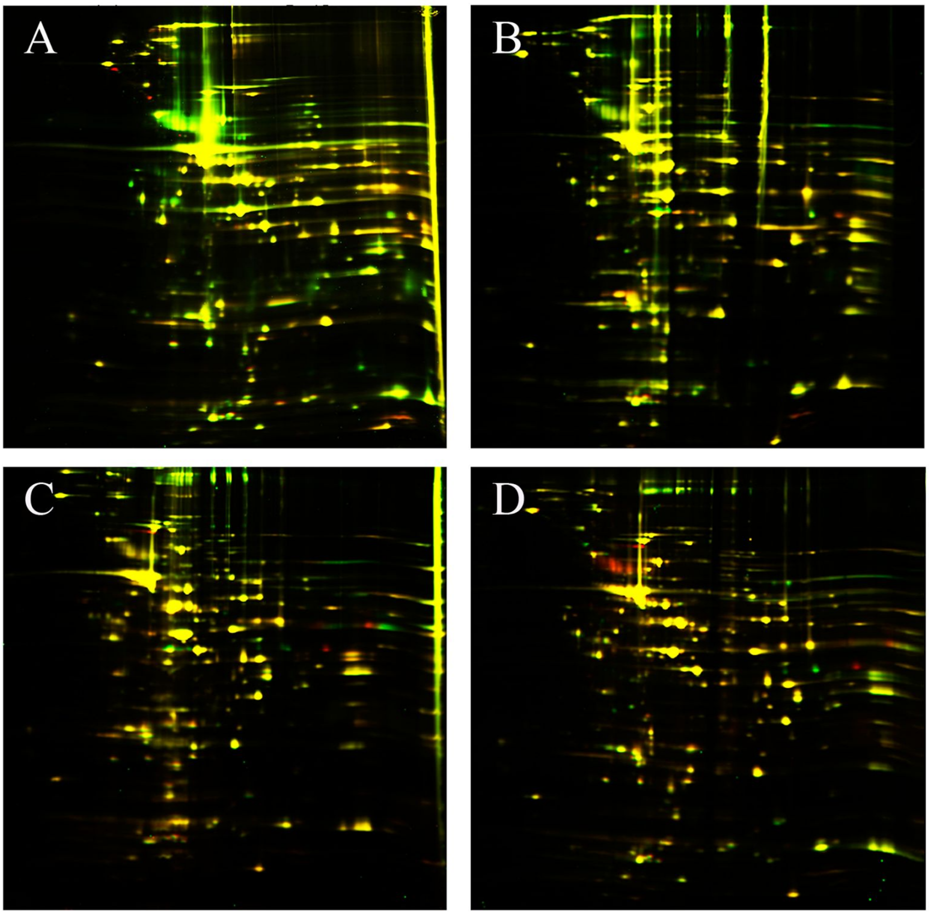

S4 Figure. 2-D DIGE analysis of passages of *M. gallisepticum* isolated from HD3 cells after 24 h of infection.

Intracellular *M. gallisepticum* stained Cy3 (red) and lab strain *M. gallisepticum S6* stained Cy5 (green). X-axis - pI ranging from 3 to 10 from left to right, Y-axis – molecular weight from 12 kDa to 150 kDa from down to up. (A) second passage. (B) sixth passage. (C) twelfth passage. (D) sixteenth passage.

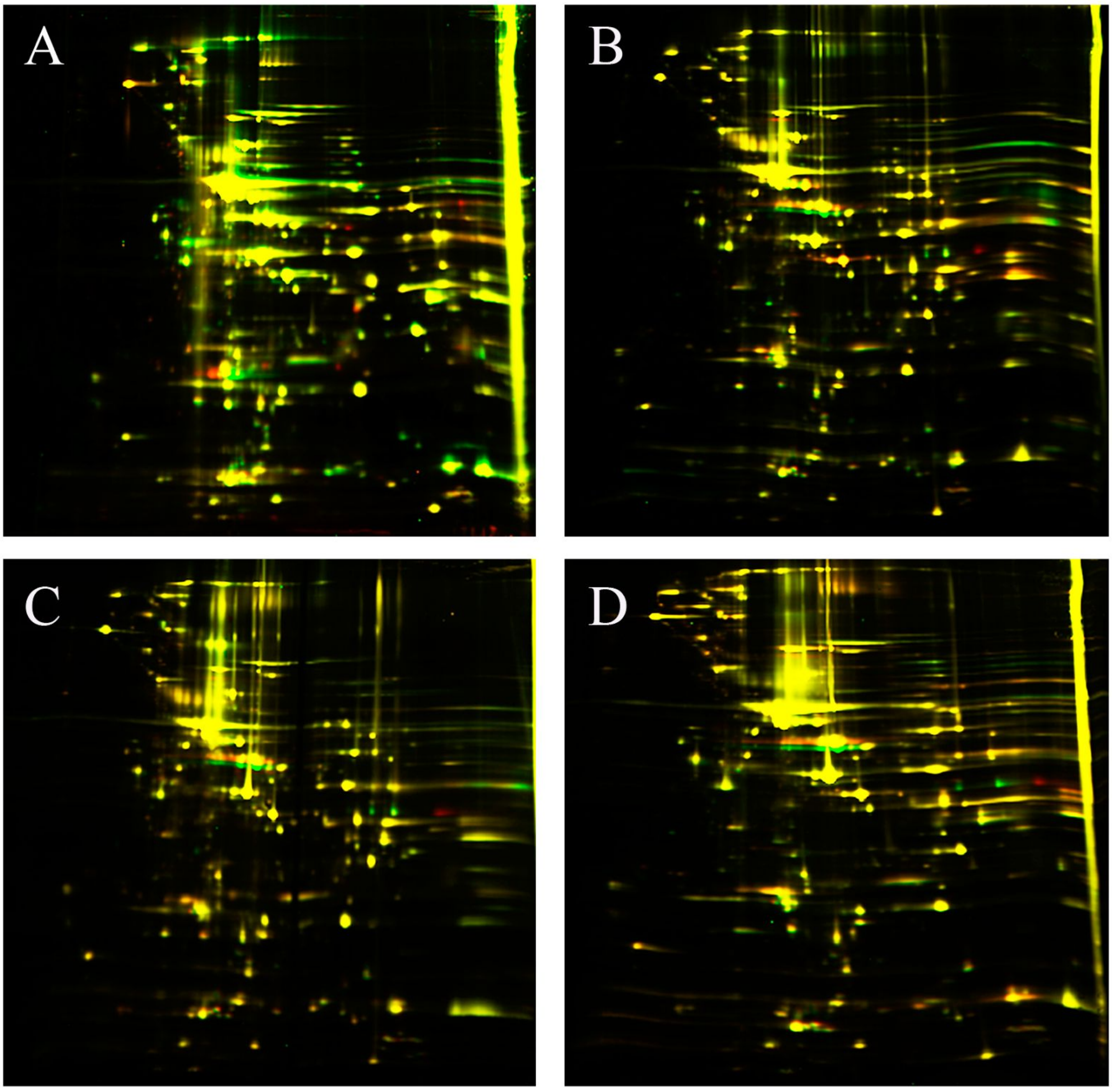

S5 Figure. 2-D DIGE analysis of passages of *M. gallisepticum* isolated from HD3 cells after chronic infection (7 weeks).

Intracellular *M. gallisepticum* stained Cy3 (red) and lab strain *M. gallisepticum S6* stained Cy5 (green). X-axis - pI ranging from 3 to 10 from left to right, Y-axis – molecular weight from 12 kDa to 150 kDa from down to up. (A) second passage. (B) sixth passage. (C) twelfth passage. (D) sixteenth passage.

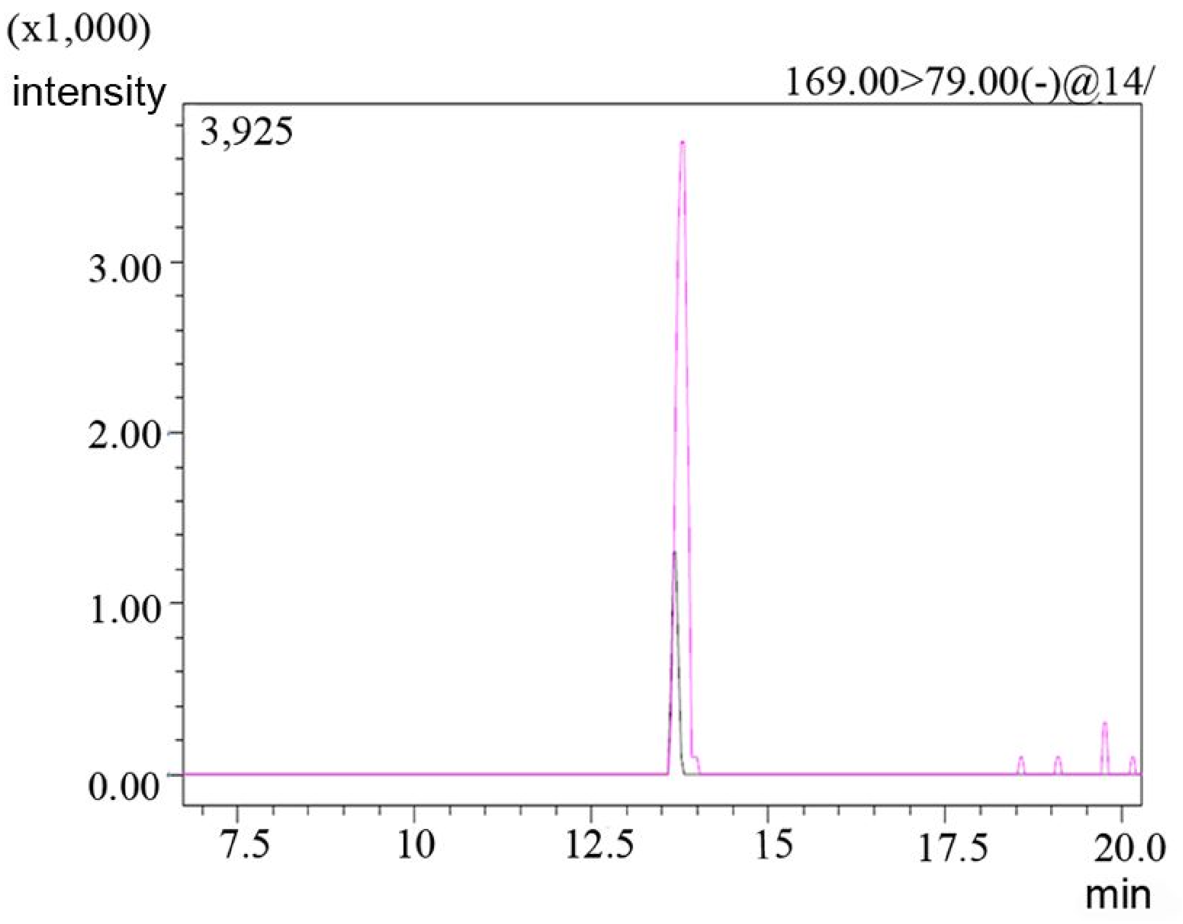

S6 Figure. Comparative MRM chromatograms for DHAP/G-3-P transition in two mycoplasma samples.

Overlay of MRM DHAP/G-3-P transitions for control laboratory *M. gallisepticum* (indicated in black) and *M. gallisepticum* isolated from mES cells after acute infection (indicated in pink) samples. Peak of DHAP/G-3-P was identified by transition 169.1/79.1 at the retention time 13.7 min.

Supplementary tables

Table S2. Changes in Vlh-hemagglutinins in *M. gallisepticum* isolated from three different cell lines after 24 h of infection compared to the control laboratory strain *M. gallisepticum S6* according MRM-analysis.

Protein name	ORF Name	Protein level (Fold-change)		
		M. gallisepticum **from HD3**	***M. gallisepticum*** **from HeLa**	***M. gallisepticum*** **from mES**
VlhA.3.07	GCW_91181	2,3	2,6	2,0
VlhA.1.01	GCW_01160	2,4	2,4	2,3
VlhA.5.02	GCW_01920	2,7	2,9	2,4
VlhA.1.01	GCW_01195	2,5	2,3	2,5
VlhA	GCW_02390	1,6	1,4	3,3
VlhA.3.03	GCW_01145	2,6	3,1	3,6
VlhA.1.01	GCW_01940	4,1	3,6	3,9
VlhA.2.02	GCW_02615	0,7	0,8	0,5
VlhA.3.08	GCW_01170	0,8	0,8	0,8
VlhA.1.01	GCW_01930	0,7	0,7	0,8
VlhA.3.03	GCW_03350	1,4	1,2	0,7
VlhA	GCW_92454	0,8	0,7	9,6
VlhA.1.01	GCW_01155	No change	No change	0,4
VlhA.1.05	GCW_02430	No change	No change	0,6
VlhA.2.01	GCW_02605	No change	No change	0,6
VlhA	GCW_92453	No change	No change	1,3

VlhA.3.06	GCW_03370	No change	0,8	1,4
VlhA	GCW_03335	No change	1,2	2,5
VlhA.1.02	GCW_02405	0,8	No change	0,2
VlhA.1.01	GCW_01175	1,2	No change	0,4
VlhA	GCW_92396	0,8	No change	0,6

The data represent the mean (±SD) of three independent experiments.

Table S3. Changes in Vlh-hemagglutinin in *M. gallisepticum* isolated from three different cell lines after chronic infection in HD3 cells compared to control laboratory strain *M. gallisepticum S6* according MRM-analysis.

**Protein name**	**ORF Name**	**Protein level (Fold-change)[a]**			
		M. gallisepticum	***M. gallisepticum* from HD3**	***M. gallisepticum* from HeLa**	***M. gallisepticum* from mES**
VlhA.1.01	GCW_01195	1,9	1,2	1,9	1,5
VlhA.3.03	GCW_03350	0,4	0,6	0,5	0,5
VlhA	GCW_03335	50,7	No change	No change	No change
VlhA.1.01	GCW_01160	14,1	No change	No change	No change
VlhA.1.01	GCW_01155	No change	0,3	0,3	0,4
VlhA.1.01	GCW_02440	No change	0,5	0,3	0,4
VlhA.3.08	GCW_01170	No change	No change	0,6	No change
VlhA.1.01	GCW_02390	No change	0,1	No change	No change
VlhA	GCW_93361	No change	0,3	No change	No change
VlhA.3.06	GCW_03370	No change	No change	No change	0,5
VlhA	GCW_91176	No change	No change	No change	0,5
VlhA	GCW_92454	No change	No change	No change	0,6

VlhA.1.01	GCW_01175	No change	No change	No change	1,6
VlhA.3.03	GCW_01145	0,5	No change	No change	0,6
VlhA	GCW_92456	2,1	No change	No change	0,5
VlhA	GCW_93371	0,6	No change	0,6	No change

[a] combined data for 19-day and 7-week chronic infections. The data represent the mean (±SD) of three independent experiments.

Table S4. Comparative genomic analysis of *M. gallisepticum* isolated from HD3 cells after acute (24h) and chronic (7 weeks) infections with laboratory strain of *M. gallisepticum S6.*

ORF Name	Gene name	Type of infection	Position	Ref	MIEC	Type of SNP
upstream of GCW_01165 in VlhA cluster 1		acute, chronic	152250	A	T,G	
GCW_01960	VlhA.1.01 cluster 2	chronic	465075	A	G	nonsyn
			465121	C	A,G	nonsyn
			465125	T	A	syn
			465131	A	T,G	nonsyn
			465137	G	A	syn
			465154	C	G	nonsyn
			465159	G	C	nonsyn
			465166	T	C	syn
			465216	T	A	nonsyn
upstream of GCW_02455 in VlhA cluster 3		chronic	588982	G	A	

upstream of GCW_03335 in VlhA cluster 4		acute, chronic	801100	T	C	
GCW_03335	VlhA cluster 4	acute	800656	T	C,A	nonsyn
GCW_93371		acute, chronic	816612	G	T	nonsyn
			816905	A	T	syn
			817733	A	G	syn
			818171	C	A,G	nonsyn
		acute	817621	G	T,A	nonsyn
		chronic	818179	C	T,G	nonsyn
			818184	T	G	nonsyn
			818186	T	A	nonsyn
			818187	G	T	syn
			818188	G	T	nonsyn
			818193	G	C	syn
			818194	G	C	nonsyn
			818202	T	A	nonsyn
			818225	T	G	nonsyn
			818226	G	T,A	nonsyn
			818228	A	G	nonsyn
			818255	T	C,A	nonsyn
upstream of GCW_93372 in VlhA cluster 4		acute	818493	T	G	
upstream mobile element protein		acute	348358	A	T	

(GCW_91455)						
upstream mobile element protein (GCW_90633)		chronic	152250	A	T,G	
GCW_00395	23S ribosomal RNA	chronic	318386	G	A	
GCW_01395	asparaginyl-tRNA synthetase	chronic	332883	G	T	syn
GCW_01520	M42 glutamyl-aminopeptidase family protein	chronic	359323	T	C	syn
GCW_01520	M42 glutamyl-aminopeptidase family protein	chronic	359324	A	C	nonsyn
GCW_92037	Phenylalanyl -tRNA synthetase (PheRS) beta chain core domain	chronic	483621	A	G,T	nonsyn
GCW_03035	30S ribosomal protein S12	chronic	723019	T	C	syn
GCW_03045	hypothetical protein DUF3682, eukaryotic protein	chronic	724257	G	C	nonsyn

MIEC - mycoplasma isolated from eukaryotic cells; **Ref** - references strain of *M. gallisepticum S6*; **syn** – synonymous; **nonsyn** – nonsynonymous.

S5 Table. Identified metabolites of *M. gallisepticum* isolated from mES after acute infection compared control laboratory *M. gallisepticum S6* strain.

Compound name	Compound ID	I1	I2	Fold I1/I2
negativ mode				
**Phosphoenolpyruvate**	C00074	38165	nd	up
**NAD+**	C00003	63736,4	53433	1,2
**Glycerol-P**	C00093	5343,1	nd	up
**Threonine**	C00188	5343,1	6511	0,8
**arginine**	C00062	25394,6	31834	0,8
**6-Phospho-D-gluconate**	C00345	20536	27334	0,8
**CMP**	C00055	3134,8	3592	0,9
**dGMP**	C00362	9769,9	2467	4,0
**Phosphoenolpyruvate**	C00074	38165	5613	6,8
**Dihydroxy-aceton-P/G-3-P**	C00111	70606,1	8756	8,1
**Erythrose-4-P-13C4**	C00279	nd	4482	down
positiv mode				
**Adenosine**	C00559	5230114,6	2016040	2,6
**Xanthosine**	C01762	1499131,4	417210	3,6
**Guanosine**	C00387	4476678	1250565	3,6
**Cytidine**	C00475	614414	343213	1,8
**Tryptophan**	C00078	185493,8	105235	1,8
**FAD**	C00016	18104,45	119922	0,2
**Adenine**	C00147	415332,1	197462	2,1
**Guanine**	C00242	1267841,3	1816768	0,7
**Lysine**	C00047	3932105,1	2999007	1,3
**NAD**	C00003	3867,5	67579	0,1
**UMP**	C00105	nd	3640	down
**AMP**	C00020	nd	47661	down
**Leucine**	C00123/C00407	942927,1	485683	1,9
**Valine**	C00183	1250108,6	670212	1,9

**Phenilalanine**	C00079	1349011,2	1043367	1,3
**Proline**	C00148	197968,4	3842416	0,1
**Arginine**	C00062	30710530,6	22406675	1,4
**Citrulline**	C00327	18786,7	78267	0,2
**Alanine**	C00041	24945,8	122195	0,2
**Threonine**	C00188	nd	24209	down
**Methionine**	C00073	400769,9	229827	1,7
**NADH**	C00004	nd	10920	down
**Nicotinamide**	C00153	65589,4	88773	0,7
**Glutamine-13C2**	C00064	679818,1	1044764	0,7
**AMP**	C00020	nd	19793	down

nd, not detectable; **I1 (test),** the value of the average normalized integrated intensity of the peak in the group of test samples (*M. gallisepticum* isolated from mES after acute infection); **I2(control),** the value of the average normalized integrated intensity of the peak in the group of control samples (control laboratory *M. gallisepticum S6* strain); **Fold I1/I2,** the ratio of the average integrated intensities. The data represent the mean (±SD) of three independent experiments.

S6 Table. Parameters of MSMS identification of metabolites.

**Compound name**	**ion transition**	**RT,min**	**Event number**
Fumarate	115.00>71.00	1,5	3:MRM(-)
Orotate	155.00>111.00	11,05	10:MRM(-)
Dyhydroorotate	157.00>113.00	11,5	11:MRM(-)
Mannitol	181.05>71.00	11,44	2:MRM(-)
Mannitol2	181.05>59.00	11,44	2:MRM(-)
Erythrose-4-P-1	199.00>97.00	14,1	21:MRM(-)
Phenylpyruvate	163.00>91.00	11,654	12:MRM(-)
Trehalose	341.00>179.00	12,279	35:MRM(-)
UDP-N-acetyl-glucoseamine	606.00>385.00	12,657	31:MRM(-)
Succinate	117.00>73.00	12,8	4:MRM(-)

Thymidine	241.00>125.00	1,95	1:MRM(-)
Malate	133.00>115.00	13,6	7:MRM(-)
a-ketoglutarate	145.00>101.00	12,5	9:MRM(-)
Gluconate	195.00>129.00	13,3	20:MRM(-)
Deoxyribose-P	213.00>79.00	13,9	22:MRM(-)
Ribose-5P1	229.00>138.90	13,9	22:MRM(-)
Ribose-5P2	229.00>96.90	13,9	22:MRM(-)
NAD^+	662.00>540.00	13,506	1:MRM(-)
Phosphoenolpyruvate	167.00>79.00	13,975	13:MRM(-)
Glycerol-P	171.00>79.00	13,778	15:MRM(-)
Glycerol-P	171.00>96.95	13,778	15:MRM(-)
Glycerol-P-13C3	174.00>79.00	13,778	15:MRM(-)
Glycerol-P-13C3	174.00>96.95	13,778	15:MRM(-)
PRPP	389.00>291.00	14,5	37:MRM(-)
dTDP	401.00>159.00	14,3	39:MRM(-)
ATP	506.00>408.00	13,838	47:MRM(-)
D-hexose-6-P	259.00>79.00	13,892	24:MRM(-)
D-hexose-6-P	259.00>199.00	13,892	24:MRM(-)
Threonine	118.00>74.00	13,7	5:MRM(-)
ADP	426.00>134.00	14,322	42:MRM(-)
dGDP	426.00>159.00	13,9	43:MRM(-)
Oxidized glutatione	611.10>306.05	14	49:MRM(-)
2-phosphoglycerate	185.00>97.00	13,9	18:MRM(-)
N-acetyl-glucoseamine-P 1	300.00>79.00	14,185	27:MRM(-)
NADP	742.00>620.00	14,487	33:MRM(-)

UDP	403.00>305.00	12,3	41:MRM(-)
Arginine	173.10>131.00	19,329	16:MRM(-)
Arginine 13C6	179.10>136.00	19,329	16:MRM(-)
Glutamate 12	146.05>128.05	13,6	16:MRM(-)
Glutamate 13c5	151.05>133.05	13,6	16:MRM(-)
Glucose 1	179.05>113.05	11,3	16:MRM(-)
Glucose 2	179.00>89.00	11,3	16:MRM(-)
Glucose 13c61	185.05>118.05	11,3	16:MRM(-)
Glucose 13c6 2	185.05>92.00	11,3	16:MRM(-)
Citrate	191.00>111.00	19,2	19:MRM(-)
Carbomail-P	140.00>79.00	25,01	8:MRM(-)
Glycerate	105.00>75.00	12,6	51:MRM(-)
Pyruvate	87.00>43.00	9,8	51:MRM(-)
Pyruvate13C	90.00>45.00	9,8	51:MRM(-)
Tryptophane1	203.10>116.05	12,5	51:MRM(-)
Tryptophane2	203.10>142.05	12,5	51:MRM(-)
Oxaloacetate	131.00>87.00	13,2	6:MRM(-)
Citrulline	174.00>131.00	14,1	17:MRM(-)
Uridine	243.00>200.00	2,5	23:MRM(-)
Glucosamine-P-(1)	258.05>78.95	12,5	25:MRM(-)
Glucosamine-P-(2)	258.05>96.95	12,5	25:MRM(-)
6-P-D-gluconate	275.00>97.00	4,59	26:MRM(-)
UDP-D-glucose	565.00>323.00	12,6	28:MRM(-)
PPGPP	602.00>504.00	24,5	30:MRM(-)
NADH	664.00>408.00	15,4	32:MRM(-)

Fructose-1,6-PP	339.00>97.00	13,8	36:MRM(-)
dCDP	386.00>159.00	10,3	38:MRM(-)
CDP	402.00>159.00	14	40:MRM(-)
IDP	427.00>159.00	14	44:MRM(-)
GDP	442.00>159.00	14	45:MRM(-)
FMN	455.00>213.00	10,2	46:MRM(-)
CMP	306.05>143.05	13,3	48:MRM(-)
Acetyl-CoA-2C13	810.10>408.00	13,6	50:MRM(-)
Acetyl-CoA1	808.10>408.00	13,6	50:MRM(-)
Acetyl-CoA2	808.10>461.05	13,6	50:MRM(-)
Acetyl-P	139.00>79.00	13,8	50:MRM(-)
Acetyl-P-2C13	141.00>79.00	13,8	50:MRM(-)
UTP	483.00>159.00	14	1:MRM(-)
CTP	482.00>159.00	14,1	1:MRM(-)
GTP	522.00>424.00	14,1	1:MRM(-)
ITP	507.00>409.00	14,1	1:MRM(-)
dATP	490.00>159.00	14,1	1:MRM(-)
dADP	410.00>79.00	14,1	1:MRM(-)
dCMP	306.10>110.00	14,1	1:MRM(-)
dCTP	466.00>159.00	14,1	1:MRM(-)
dGMP	346.00>78.90	13,8	1:MRM(-)
dGTP	506.10>408.10	14,1	1:MRM(-)
XTP	523.00>159.00	14,1	1:MRM(-)
TTP	481.00>159.00	14,1	1:MRM(-)
NADPH	744.00>408.00	14,4	34:MRM(-)

Hexose-6-P 3	259.00>169.00	13,8	24:MRM(-)
Hexose-6-P 1	259.00>79.00	13,8	24:MRM(-)
Hexose-6-P-13C6 1	265.00>79.00	13,8	24:MRM(-)
Hexose-6-P-13C6 2	265.00>203.00	13,8	24:MRM(-)
Hexose-6-P-13C6 3	265.00>172.00	13,8	24:MRM(-)
Dihydroxy-aceton-P/G-3-P1	169.00>79.00	13,6	14:MRM(-)
Dihydroxy-aceton-P/G-3-P2	169.00>96.95	13,6	14:MRM(-)
DHAP/G-3-P-13C3 1	172.00>79.00	13,6	14:MRM(-)
DHAP/G-3-P-13C3 2	172.00>96.95	13,6	14:MRM(-)
Glycerate-1,3-PP	265.00>78.95	13,8	25:MRM(-)
Glycerate-1,3-PP-13C3	268.00>78.95	13,8	25:MRM(-)
Glycerate-3-P/Glycerate-2-P	185.00>97.00	13,8	18:MRM(-)
Glycerate-3-P/Glycerate-2-P-13C3	188.00>97.00	13,8	18:MRM(-)
Glycerate-3-P	185.00>169.00	13,8	18:MRM(-)
Glycerate-3-P-13C3	188.00>172.00	13,8	18:MRM(-)
Glycerate-2-P	185.00>141.00	13,8	18:MRM(-)
Glycerate-2-P-13c3	188.00>144.00	12,58	18:MRM(-)
PEP	167.00>79.00	13,7	13:MRM(-)
PEP 13C3	170.00>79.00	13,7	13:MRM(-)
Lactate	89.00>41.00	8,9	51:MRM(-)
Lactate-13C3	92.00>43.00	8,9	51:MRM(-)
Ac-CoA-13c2 1	810.10>408.00	13,5	50:MRM(-)
Ac-CoA-13c2 2	810.00>463.05	13,5	50:MRM(-)
Ribose-5P-13C5 1	234.00>141.00	13,9	22:MRM(-)

Ribose-5P-13c5 2	234.00>96.90	13,9	22:MRM(-)
Erythrose-4-P 2	199.00>138.90	14,1	21:MRM(-)
Erythrose-4-P-13C4 1	203.00>97.00	14,1	21:MRM(-)
Erythrose-4-P-13c4 2	203.00>141.00	14,1	21:MRM(-)
Alanine	88.05>42.00	12	5:MRM(-)
Alanine-13C3	91.00>91.10	12	5:MRM(-)
Thymidine	241.00>125.00	2	1:MRM(-)
Acetyl-Glucosamine 1	220.10>59.00	0,001	36:MRM(-)
Acetyl-Glucosamine 2	220.10>101.00	0,001	36:MRM(-)
Acetyl-Glucosamine 3	220.10>176.60	0,001	36:MRM(-)
Glutamin1	145.05>101.00	13,5	9:MRM(-)
Glutamin2	145.05>127.10	13,5	9:MRM(-)
Glutamin-13C5 2	150.05>132.10	13,5	9:MRM(-)
Glutamin3	145.05>109.05	13,5	9:MRM(-)
Glutamin-13C5 3	150.05>113.05	13,5	9:MRM(-)
Glutamate1	146.05>128.05	13,5	9:MRM(-)
Glutamate-13C51	151.05>133.05	13,5	9:MRM(-)
Glutamate2	146.05>102.05	13,5	9:MRM(-)
Glutamate-13C 2	151.05>106.05	13,3	9:MRM(-)
Glutamin4	145.10>42.00	13,5	9:MRM(-)
